# Treatment of Neuroma-induced Chronic Pain and Management of Nerve Defects with Processed Nerve Allografts

**DOI:** 10.1097/GOX.0000000000002467

**Published:** 2019-12-19

**Authors:** Ivica Ducic, Joshua Yoon, Kyle R. Eberlin

**Affiliations:** From the * Washington Nerve Institute, McLean, Va.; † Department of Surgery, The George Washington University, Washington, D.C.; ‡ Division of Plastic and Reconstructive Surgery, Massachusetts General Hospital, Harvard Medical School, Boston, Mass.

## Abstract

Symptomatic neuromas can cause significant chronic pain and negatively impact quality of life. Symptoms often persist despite narcotics and nonoperative interventions, which are largely ineffective. With the opioid crisis, treatments for chronic pain that limit narcotics are needed. Traditional surgical options may result in neuroma recurrence. Autograft reconstruction often results in donor-site morbidity. Processed nerve allografts facilitate axonal growth, nerve regeneration, and eliminate donor-site morbidity.

**Methods:**

A literature review was performed to identify studies in which chronic neuroma pain was treated with excision and processed nerve allograft reconstruction. PubMed was queried, and data from the studies were grouped into treatment effective and ineffective groups. Statistical analyses were performed on these groups, and further subgroup analysis was performed on overall change of preoperative and postoperative pain scores using a paired *t* test.

**Results:**

Seven studies fulfilled inclusion criteria yielding 42 patients. Greater than 90% of patients had improvement of pain postoperatively. The preoperative and postoperative pain scores could be determined for 40 patients. The mean preoperative score was 7.9, and the mean postoperative score was 3.54. These results were statistically significant using a paired *t* test with a *P* value of <0.001.

**Conclusions:**

Chronic pain resulting from symptomatic neuromas can be treated with neuroma excision and nerve stump reconstruction with processed nerve allograft. This obviates autograft-associated donor-site morbidity and provides a platform to potentially restore sensation to the involved nerve whenever a distal nerve end is available. Addressing the root cause is an important paradigm shift for treating symptomatic neuromas.

## INTRODUCTION

Neuromas secondary to trauma or iatrogenic injury are the result of proliferation during disorganized regeneration of an injured nerve.^1^ Symptomatic neuromas can be a significant source of chronic pain and negatively impact quality of life.^2,3^ With the current opioid crisis, treatments for chronic pain that limit the need for narcotics are needed.^4^ Neuropathic pain secondary to neuromas is often resistant to medical treatments and conventional pharmacotherapy. Only 30%–40% of patients are able to achieve adequate, durable pain control with medications alone.^5^ Nonsurgical interventions such as radio-frequency ablation are transiently effective and there is a high rate of symptom relapse, and nerve stimulators are effective beyond 3 years only about 50% of the time.^6–8^

Medical treatments fail to provide long-lasting relief for chronic neuroma pain primarily because they are symptomatic treatments and do not address the underlying etiology. Traditional surgical options have attempted to address the root cause and include neuroma excision followed by implantation of the nerve stump into bone or muscle, traction neurectomy, and nerve capping.^8–10^ Traditional surgical modalities, however, may result in aberrant nerve regeneration and have been shown to have high postoperative recurrence rates, which can cause recurrence or even exacerbation of the original pain.^2,11,12^ Other recent methods to address neuroma pain following neuroma excision also include autologous nerve reconstruction, allograft nerve reconstruction, targeted muscle reinnervation, and regenerative peripheral nerve interface.^12^ The aim of this study is to review the current available literature and examine the role of processed human nerve allograft reconstruction in the treatment of neuropathic pain following excision of symptomatic neuromas.

## METHODS

This review was guided by the Preferred Reporting Items for Systematic Review and Meta-Analyses checklist. PubMed and online literature review performed to identify currently available studies in which neuropathic pain secondary to neuromas was treated with neuroma excision and nerve allograft reconstruction. There were no limits placed on study publication date, publication status, minimum follow-up time, or design. All currently available studies including case reports were considered for inclusion. Relevant studies were identified and chosen by utilizing the search terms: neuroma, pain, allograft, and treatment. Studies were excluded if the article was not available in the English language. From the search results, the authors screened the titles or abstracts to determine relevance and study eligibility. The eligible studies were then reviewed independently by the authors and were included in the review upon reaching a unanimous consensus. The data from the included studies were compiled and grouped into treatment effective and treatment ineffective groups with effectiveness being defined as improvement in pain. Statistical analyses were performed on these groups, and further subgroup analysis was performed on overall change of preoperative and postoperative pain scores using a paired *t* test to determine percent of patients with pain improvement and magnitude of improvement. During the subgroup analysis to determine preoperative and postoperative pain scores, the pain scores for each individual patient or set of patients were reviewed individually. If there was no pain score documented for the patient or set of patients, then they were not included in the analysis.

## RESULTS

**Fig. 1.**
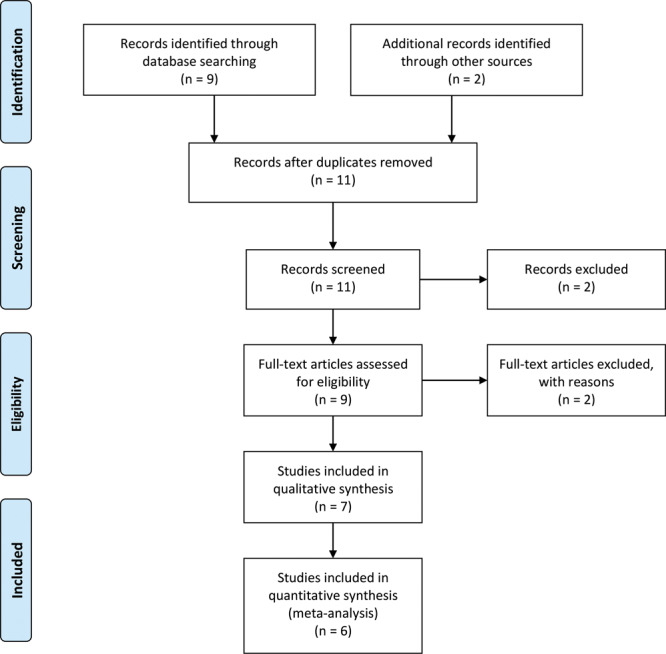
Study screening and selection algorithm.

The literature search on PubMed conducted on December 31, 2018, identified 9 total studies, and 2 additional studies were identified through other sources. After screening of titles and abstracts, 2 studies were excluded. After reviewing the 9 remaining manuscripts, 2 were excluded due to clinical irrelevance to our primary question (Fig. 1). A total of 7 studies fulfilled inclusion criteria yielding a total of 42 patients who underwent neuroma resection with concomitant use of nerve allograft.^13–19^ (Table 1) Over 90% of all patients with chronic pain resistant to conventional medical treatments, who underwent neuroma excision with allograft reconstruction responded favorably and had decreased pain. The preoperative and postoperative pain scores could be determined for 40 patients. The mean preoperative pain score was 7.9, and the mean postoperative score was 3.6. These results were found to be statistically significant (*P* < 0.001).

**Table 1.** Analyzed Studies

Study Name	n	Reconstruction Technique	Results
Souza et al^13^	22 (6 SPN, 9 sural, 5 digital, 1 DPN, 1 LPN), 7 end neuroma, 15 neuroma in continuity	-End-to-end coaptation -Polypropylene epineurial sutures	-Ordinal pain scores decrease by mean of 2.6 (range, +2 to −8) -Pain behavior scores decrease by mean 7.3 (range, +2 to −22) and percentile decrease of 24% -Pain interference decreased by mean 11.3 (range, +2 to −27) and percentile decrease 30.7%
Bibbo and Rodriguez-Colazzo^14^	4 (2 sural, 2 SPN), 3 end neuroma, 1 neuroma in continuity	-End-to-side coaptation with porcine submucosa nerve connectors -Nylon epineurial sutures	-Mean preoperative visual analog scale pain score 9.5 (range, 8–10) -Mean postoperative VAS pain score 1.25 (range, 0–2)
Rodriguez-Colazzo et al^15^	2 deep peroneal end neuroma s/p failed nerve implantation into muscle	-End-to-end coaptation with bovine collagen wrap -Nylon epineurial sutures -Implantation into bone with muscle flap coverage	-Preoperative patient 1 endorses “excruciating” pain, patient 2 rated pain 9/10 -No pain at third postoperative visit, although walking with assistance of walker
Bi et al^16^	1 abdominal wall neuroma in continuity	-End-to-end coaptation -Polypropylene epineurial sutures	-Heavy preoperative narcotic dependence, weaned from narcotics at 7 mo postoperative -Preoperative pain behavior score 92nd percentile to 94th postoperative -Preoperative pain interference score 87th percentile to 42nd postoperative
Bibbo et al^17^	11 SPN	-End-to-end coaptation with porcine submucosa nerve connectors -Nylon epineurial sutures	-Mean preoperative VAS pain score 7.9 (range, 7–9) -Mean postoperative VAS pain score 2.45
Bassilios Habre et al^18^	1 supraorbital nerve	-End-to-end coaptation with collagen nerve wrap -Nylon epineurial sutures	-Mean preoperative VAS pain score 8 -No postoperative pain at 1 y
Freniere et al^19^	1 radial and ulnar digital nerve end neuromas	-End-to-end coaptation	-Preoperative VAS pain score 9 -Postoperative VAS pain score 2 at 6 mo

DPN, deep peroneal nerve; LPN, lateral plantar nerve; s/ p, status post; SPN, superficial peroneal nerve; VAS, visual analog scale.

## DISCUSSION

It has been shown that treating chronic neuroma pain with traction neurectomy alone, now considered an outdated technique, is associated with a high rate of symptomatic recurrence.^11^ Subsequent efforts to control neuroma-induced chronic pain and neuroma recurrence focused on the implantation of the proximal nerve stump. Thus, several techniques evolved over time following neuroma excision, which include implantation of the proximal nerve stump to bone, vein, and/or most commonly to muscle.^9,10,20–22^ One of the potential reasons for an improved outcome observed with these techniques was that this intervention has helped to facilitate a physiologic environment to promote proper axonal regeneration and limit aberrant nerve growth. Still, these treatment methods do not seem universally successful as symptomatic neuromas can recur. Contrary to these traditional passive methods, there has been an ongoing paradigm shift where now more active treatments are being applied.^23^ When choosing between different treatment modalities, the primary determinants that will guide the decision are the presence and size of a nerve gap and the existence of a distal nerve ending.

If there is no distal end present, then emerging data for targeted muscle reinnervation and regenerative peripheral nerve interface demonstrate improvement in outcomes.^12,24–26^ Similar promising outcomes have been observed with capping of the nerve ends, which is principally directed toward minimizing symptomatic neuroma recurrence.^27,28^ We believe that the principle of guided nerve regeneration is an important tenet in facilitating controlled, directed neural regrowth while minimizing risk of neuroma recurrence and therefore improving patient outcomes. Still, prospective, larger, and controlled studies are needed to validate such considerations.

With the presence of a distal nerve end, if there is no nerve gap, then a tensionless direct coaptation is the procedure of choice.^29^ However, in our experience with neuroma excision, adequate resection to healthy tissue almost always results in a sufficiently large nerve gap that requires a bridging medium for tensionless reconstruction.

One common technique to bridge a nerve gap is the use of hollow tube conduits. There are two 510(k)-cleared generations of conduits. First-generation conduits are synthetic, whereas second-generation conduits are composed of biologic materials such as porcine intestinal submucosa.^30,31^ A review performed by Safa and Buncke^32^ found that conduits performed well in gaps under 6 mm, but beyond this length, the reliability declined rapidly and outcomes were significantly less consistent.

Considering these deficiencies, autologous nerve grafts are commonly used and are the traditional gold standard in nerve gap reconstruction.^33–35^ Autografts have generally favorable outcomes in nerve gap reconstruction, but they have associated drawbacks including additional incisions, longer operative time, and limited availability of autologous nerve tissue. In addition, donor-site morbidity includes wound healing issues, neuroma formation, and permanent loss of sensation.^33–40^ In the authors’ experience, most patients with symptomatic neuromas will not agree to autologous nerve grafting given the risk of neuropathic pain at the donor site. Creating a permanent sensory deficit with potential paresthesia within a donor-nerve distribution to reconstruct another damaged sensory nerve that is generating chronic pain is a suboptimal situation. Still, it is increasingly clear that management with reconstructive solutions is a desired way to address symptomatic neuroma pain due to nerve injuries.^41^

A search for a more appropriate bridging material directed us to the use of processed human nerve allograft (Avance Nerve Graft; AxoGen Corporation, Alachua, Fla.) intended for the surgical repair of peripheral nerve discontinuities to support regeneration across the defect. It is an extracellular matrix scaffold from donated human peripheral nerve tissue that has been predegenerated, decellularized, and sterilized. The decellularization and sterilization of the allograft minimize the risk of immune rejection, which eliminates the need for immunosuppressive therapy and also maintains the native architecture of the nerve including the extracellular matrix proteins (laminin, fibronectin, and glycosaminoglycans).^42,43^ These proteins, in addition to the native microscopic structure, provide architecture for guided regrowth.^42–47^ A growing body of evidence has demonstrated that processed nerve allografts are safe and have comparable results to autografts without the associated donor-site morbidity in nerve gaps up to 70 mm.^47^ The clinical outcomes of nerve allografts in comparison to the other modalities are attributed to the structural preservation of the nerve architecture and proteins in the nerve microenvironment.^45,48–51^ As a result, autograft and allograft reconstruction are acceptable techniques to bridge a nerve gap >6 mm with comparable results.

**Fig. 2.**
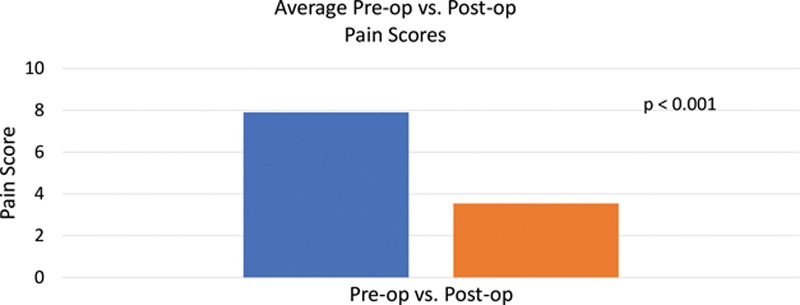
Preoperative versus postoperative pain scores after nerve allograft reconstruction following neuroma excision for chronic neuroma pain.

The most frequently used metric for pain measurement in the analyzed studies is the visual analog scale. Bi et al^16^ and Souza et al^13^ elected to use the National Institutes of Health–developed Patient Reported Outcomes Measurement Information System questionnaires for Pain Behavior and Pain Interference. The Patient Reported Outcomes Measurement Information System scores allow for a multifaceted analysis of not only a patient’s perceived pain, but also how the level of pain translates into day-to-day activities. Regardless of the tool used, all analyzed studies individually demonstrated overall clinical improvement of chronic pain after neuroma resection and allograft reconstruction. Together, the studies demonstrated a 90% favorable response rate among all patients with a statistically significant improvement in pain scores (Fig. 2). The majority of the studies, however, did not examine how medical/nonsurgical pain management changed postoperatively; thus, it is difficult to fully assess whether the decrease in pain translated to less medication use. Bi et al^16^ were able to demonstrate the ability of patients to be weaned from narcotics completely in a matter of months following allograft reconstruction. Rodriguez-Colazzo et al^15^ demonstrated a clinically significant change by showing a resolution of pain that functionally limited ambulation.

The studies were collectively grouped and analyzed under the umbrella technique of nerve allograft reconstruction, but there are several important nuances and distinctions. The majority of the studies reconstructed the nerve in an end-to-end fashion; however, Bibbo^14^,^17^ utilized an end-to-side technique and yielded comparable results. Whether or not there are any differences in outcomes between these techniques will require further study; however, the principle underlying these two different techniques remains the same: coordinated, guided regrowth leads to more normal nerve regeneration and minimizes neuroma formation.^29,51,52^ Specifically, nerve reconstruction with an allograft after neuroma excision allows for the healthy nerve tissue to regrow in a fashion akin to traditional neurotization procedures. Nerve regrowth is not only better directed, but when nerve continuity is established, it also allows for potential neurotization of the nerve’s original target, thus allowing for an added benefit of sensory restoration in addition to improvement of pain.

Among the end-to-end reconstructions, nerve allografts were typically used to restore continuity of a single nerve. An alternative technique applied by Bibbo in a separate study connected two separate nerves (superficial and deep peroneal nerves) in an end-to-end fashion and effectively relocated the nerve. Patient’s pain improved, satisfaction remained high, and clinical functional impairment was reported low. This technique remains an option to address pain related to superficial peroneal or deep peroneal nerve neuromas and may further be applied to different nerves in proximity. This technique, however, would remove the possibility of neurotization and reinnervation of a nerve’s original target. Similarly, Freniere et al^19^ also performed relocation nerve grafting for a digital neuroma. The patient had pain that was caused by a finger amputation stump neuroma that was successfully managed by resection and subsequent nerve relocation transfer into the proximal web space.

There are 3 broad distinct nerve grafting techniques used to repair the nerve after neuroma removal with the goal of reducing the risk of neuroma recurrence. The first modality is reconstruction of the nerve gap, where the original course of the nerve is restored. The second is an end-to-side nerve repair where the damaged nerve is connected to the side of an undamaged nerve, to continue its regeneration. The last is a relocation nerve grafting, in which the axons are redirected through the nerve graft to a more favorable target location. When possible, nerve gap reconstruction should be pursued to take advantage of the potential benefit of sensory restoration and would likely be possible when excising neuromas in continuity. When nerve gap reconstruction is not possible, reported end-to-side or relocation nerve grafting procedure could be pursued; however, there are no enough data to determine if there are any clinical advantages or differences between these 2 techniques. Until such data become available, the choice should be made in conjunction with the patient and at the surgeon’s discretion.

Further subanalysis of the available data from Bi et al^16^ showed that not only did the neuroma pain resolve, but also that the original sensory area of the nerve returned over time. However, restoring continuity of a nerve is not always possible and other means of restoring target area sensation or function can be performed. Specifically, Bassilios Habre et al^18^ demonstrated that the principles utilized in direct muscle neurotization can be applied to an allograft in restoring sensation for the supraorbital nerve after neuroma resection. Author was able to transcutaneously suture divided nerve fascicles from the allograft to the original sensory territory of the supraorbital nerve and restore sensation. The concern with this technique for neuroma recurrence would be higher given that there would be free nerve stumps in a potentially less-than-ideal environment and thus needs to be further explored with longer follow-up and additional studies.

Additionally, we analyzed the different suture types used during repairs for differences in pain scores. The use of nylon versus polypropylene sutures did not demonstrate that the suture type provided a statistically or clinically significant impact on pain scores. Although the study sample size was not large enough to demonstrate sutures differences, the literature review suggests that the nerve connector (conduit/wrap) at the recipient nerve allograft coaptation site may improve outcomes and reduce pain.^29^ The likely mechanism is by minimizing axonal escape or misdirection at the coaptation site and thus neuroma recurrence.^27,50–52^ Due to their permeability, pliable nature, potential for revascularization, and translucency, the porcine intestinal submucosa nerve connector offers additional practical advantages over other hollow tubes.

The body of literature regarding allograft nerve gap reconstruction following neuroma excision in the treatment of chronic neuroma pain is still limited at this time given the relative novelty of these combined procedures, which is a limitation of this study. Because of the paucity of data, case reports and case series were evaluated in this study, which may introduce bias. However, the evidence available strongly suggests that allograft nerve stump reconstruction is a viable means of addressing nerve defects following neuroma excision for chronic neuroma pain and minimizing neuroma recurrence. Another limitation is that there is no direct comparison to other techniques such as autologous or hollow tube conduit reconstruction in the surgical management of symptomatic neuroma pain treatment. The data are also relatively limited for those techniques at this time. Future studies should more clearly analyze nerve reconstruction and postoperative pain management outcomes and would be standardized to allow for a meta-analysis. Future studies should also be aimed at parsing out the effectiveness and indications for the 3 aforementioned distinct nerve grafting reconstruction techniques in various clinical scenarios. Aside from these objective limitations, authors in these 7 studies successfully demonstrated various allograft applicability options to repair peripheral nerve after neuroma excision to remove patient pain offering a solution for some challenging situations where other conventional treatments are often suboptimal.

## CONCLUSIONS

Chronic pain caused by symptomatic neuroma can be improved with neuroma excision with subsequent restoration of neural continuity with processed nerve allograft nerve repair. In contrast to other surgical pain management strategies, this approach aims to minimize neuroma recurrence, prevents donor-site morbidity, serves to potentially restore sensation and function to the affected area, and offers a surgical alternative to the management of neuroma pain. As such, it represents an important treatment paradigm shift in the treatment of symptomatic neuromas.
